# Growth hormone insensitivity with immune dysfunction caused by a STAT5B mutation in the south of Brazil: evidence for a founder effect

**DOI:** 10.1590/1678-4685-GMB-2016-0231

**Published:** 2017-06-05

**Authors:** Renata C. Scalco, Fernanda T. Gonçalves, Hadassa C. Santos, Mari M. S. G. Cardena, Carlos A. Tonelli, Mariana F. A. Funari, Rosana M. Aracava, Alexandre C. Pereira, Cintia Fridman, Alexander A. L. Jorge

**Affiliations:** 1 Unidade de Endocrinologia Genética (LIM25), Faculdade de Medicina da Universidade de São Paulo (FMUSP), São Paulo, SP, Brazil; 2 Unidade de Endocrinologia do Desenvolvimento, Laboratório de Hormônios e Genética Molecular (LIM42), Faculdade de Medicina da Universidade de São Paulo (FMUSP), São Paulo, SP, Brazil; 3 Departamento de Medicina Legal, Ética Médica e Medicina Social e do Trabalho, Laboratório de Imunohematologia e Hematologia Forense (LIM40), Hospital das Clínicas da Faculdade de Medicina da Universidade de São Paulo (HC da FMUSP), São Paulo, SP, Brazil; 4 Laboratório de Cardiologia Genética e Molecular, Instituto do Coração (InCor), Faculdade de Medicina da Universidade de São Paulo, São Paulo, SP, Brazil; 5 Universidade do Extremo Sul de Santa Catarina, Criciúma, SC, Brazil

**Keywords:** founder effect, growth hormone insensitivity, immune dysfunction, STAT5B

## Abstract

Homozygous *STAT5B* mutations causing growth hormone insensitivity with immune dysfunction were described in 10 patients since 2003, including two Brazilian brothers from the south of Brazil. Our objectives were to evaluate the prevalence of their *STAT5B* mutation in this region and to analyze the presence of a founder effect. We obtained DNA samples from 1,205 local inhabitants, 48 relatives of the homozygous patients and four individuals of another affected family. Genotyping for *STAT5B* c.424_427del mutation and for two polymorphic markers around it was done through fragment analysis technique. We also determined Y-chromosome and mtDNA haplotypes and genomic ancestry in heterozygous carriers. We identified seven families with *STAT5B* c.424_427del mutation, with 33 heterozygous individuals. The minor allelic frequency of this mutation was 0.29% in this population (confidence interval 95% 0.08-0.5%), which is significantly higher than the frequency of other pathogenic *STAT5B* allele variants observed in public databases (p < 0.001). All heterozygous carriers had the same haplotype present in the homozygous patients, found in only 9.4% of non-carriers (p < 0.001), supporting the existence of a founder effect. The Y-chromosome haplotype, mtDNA and genomic ancestry analysis indicated a European origin of this mutation. Our results provide compelling evidence for a founder effect of *STAT5B* c.424_427del mutation.

## Introduction

STAT5B (OMIM 604260) is a key protein in the signaling pathway of substances that activate type I class cytokine receptors, such as growth hormone (GH), prolactin and interleukin 2 (IL2). Homozygous mutations in *STAT5B* gene cause growth hormone insensitivity (Hwa *et al.*, 2011; Scalco *et al.*, 2013), a syndrome characterized by the inability of target tissues to respond to growth hormone. The classic phenotype includes the same clinical signs found in growth hormone deficiency (for example, severe short stature, saddle nose and abdominal obesity), low IGF1 levels and normal to high basal and stimulated GH levels. STAT5B deficient patients also present signs of immune dysregulation, such as recurrent infections, severe eczema and lymphoid interstitial pneumonia, a condition that is often associated to autoimmune diseases and that may evolve to progressive pulmonary fibrosis (Hwa *et al.*, 2011; Scalco *et al.*, 2013). These immune dysfunctions are partly due to a compromised IL2 signaling, since this interleukin is an essential factor in regulatory T-cells development and T-cells activation (Jenks *et al.*, 2013). Furthermore, heterozygous carriers were also shown to be significantly shorter (~ 3.9 cm) than population-matched controls, suggesting a mild effect of *STAT5B* haploinsufficiency (Scalco *et al.*, 2015).

Since the first case report in 2003, a total of ten patients homozygous for seven different mutations in *STAT5B* were described (Kofoed *et al.*, 2003; Hwa *et al.*, 2005, 2007; Bernasconi *et al.*, 2006; Vidarsdottir *et al.*, 2006; Pugliese-Pires *et al.*, 2010; Scaglia *et al.*, 2012), including two Brazilian brothers homozygous for *STAT5B* c.424_427del mutation (Pugliese-Pires *et al.*, 2010). These brothers were born at Criciúma, a city located in the south of Brazil (at 28°40′39″ S 49°22′11″ W), with an estimated population of 204,667 individuals according to the Brazilian Institute of Geography and Statistics (IBGE) 2014 census. Their parents, heterozygous for the same mutation, denied consanguinity. Moreover, they reported that an unrelated family in the same city had two kids with similar clinical findings, deceased as a consequence of respiratory failure.

The absence of known consanguinity between the boys' parents and the presence of other patients with similar conditions in the city led us to hypothesize an increased prevalence of this mutation in the region of Criciúma and a possible founder effect explaining these cases. Furthermore, studying the prevalence of *STAT5B* c.424_427del *STAT5B* mutation could enable the prediction of recurrence of homozygosity for it.

## Subjects and Methods

### Subjects

The local ethics committee approved this study and all individuals gave their written informed consent. We obtained oral swabs samples from 1,205 adult individuals whose parents or grandparents were born in Criciúma or in the neighboring cities. These subjects were randomly selected among the patients from a health care center in Criciúma. This number of individuals allowed us to evaluate a 0.2% frequency of *STAT5B* c.424_427del allele with 0.99 statistical power. Additionally, we obtained blood samples from 48 relatives of the homozygous boys and from four individuals of the other family possibly affected by a *STAT5B* mutation, the parents and two sisters of the deceased children. We also genotyped two polymorphic markers around this mutation in all heterozygous carriers and in 53 randomly selected non-carriers to evaluate the presence of a founder effect. With the same purpose, we determined Y-chromosome and mtDNA haplotypes and genomic ancestry in one heterozygous carrier from each family affected by *STAT5B* c.424_427del mutation.

### *STAT5B* genotyping

We genotyped all samples for *STAT5B* c.424_427del mutation. Genomic DNA was isolated from oral swabs or from peripheral blood leukocytes using standard techniques. Since *STAT5B* c.424_427del mutation is characterized by the loss of four nucleotides, it was possible to detect it through fragment analysis technique. The region around this mutation was amplified by polymerase chain reaction (PCR) using the following primers: forward 5′ CTCAGTCTTCCTCCCATTCG 3′ and reverse 5′ GGCTCTCCTGGTACTGGA 3′ (amplification conditions are available on request). The products were submitted to capillary electrophoresis in the ABI PRISM 3130 sequencer and then analyzed by the GeneScan^®^ software, both from Applied Biosystems (Foster City, CA, USA).

### *STAT5B* c.424_427del allele frequency analysis

We compared *STAT5B* c.424_427del allele frequency to the frequency of other pathogenic allele variants identified in public population databases: Exome Aggregation Consortium (ExAC - http://exac.broadinstitute.org/) and NHLBI-GO Exome Sequencing Project (ESP - http://evs.gs.washington.edu/EVS/). We considered as pathogenic only the allele variants classified as stop gain or frameshift, since none of the missense variants described in these databases was evaluated by functional studies.

### Microsatellite analysis

To evaluate if the presence of this mutation in different families was due to a founder effect, we genotyped two polymorphic markers located less than 1 centimorgan (cM) far from *STAT5B* c.424_427del mutation. These markers, D17S1801 (0.04 cM from the mutation) and D17S932 (0.83 cM from the mutation), were analyzed in the two homozygous patients, 33 heterozygous individuals from seven families harboring the same *STAT5B* mutation and 53 randomly selected non-carriers from the region. They were studied through fragment analysis technique and the PCR products were submitted to capillary electrophoresis in the ABI PRISM 3130 sequencer and then analyzed by the GeneScan^®^ software.

### Evaluation of ancestry in individuals heterozygous for STAT5B c.424_427del mutation

**Figure 1:**
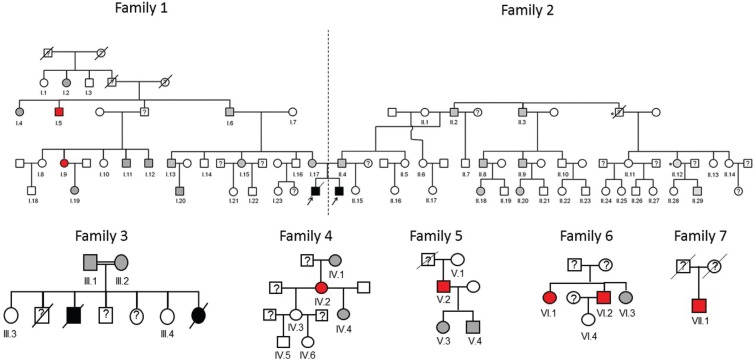
The seven identified Brazilian families with *STAT5B* c.424_427del mutation. Homozygous patients are indicated by black symbols, heterozygous carriers by gray symbols and non-carriers by white symbols. Red symbols represent the individuals identified through an active search for this mutation in the city of Criciúma. The symbol * refers to two individuals with severe pulmonary disease of unknown etiology. In family 3, the diagnosis of homozygosity for *STAT5B* c.424_427del mutation in the two deceased siblings was inferred from their clinical data and from the finding that their parents are heterozygous carriers.

We assessed genomic, Y-chromosome and mtDNA ancestry in seven individuals (6 males) carrying *STAT5B* c.424_427del mutation, one from each identified family (Figure 1 – subjects I.6, II.2, III.1, IV.2, V.2, VI.2 and VII.1).

#### Genomic ancestry analysis

Analysis of genomic ancestry was conducted using the Admixture program (Alexander *et al.*, 2009). This software estimates parameter standard errors using bootstrapping. We used an unsupervised approach for ancestry determination, with 200 bootstrap replicates (default) and k = 3 (number of parental populations assumed for the analysis). The analysis was done using 192 Ancestry Informative Markers developed by Santos HC (Santos *et al.*, 2016) and genotyped by OpenArray® Real-Time PCR (Applied Biosystems). We assumed as reference ancestral populations: Pima and Maya as Amerindians (from the Human Genome Diversity Project - HGDP); YRI (Yoruba in Ibadan, Nigeria), LWK (Luhya in Webuye, Kenya) and ASW (Americans of African Ancestry in SW, USA) as Africans (from the HapMap project); and CEU (Utah Residents (CEPH) with Northern and Western European ancestry) and TSI (Tuscan in Italia) as European (from the HapMap project).

#### Y-chromosome haplotype analysis

The set of 17 Y-STRs was amplified using the AmpFlSTR® YfilerTM kit (Applied Biosystems) which contains the markers DYS19, DYS389 I, DYS389 II, DYS390, DYS391, DYS392, DYS393, DYS385 a/b, DYS438, DYS439, DYS437, DYS448, DYS456, DYS458, DYS635, Y GATA H4. Capillary electrophoresis was done in an ABI PRISM 3130 sequencer, and sizes were assigned to the different fragments using GeneMapper® software. The nomenclature of alleles followed the recommendations of the DNA Commission of the International Society of Forensic Genetics, except for locus Y GATA H4, which was named on the basis of the allelic ladder supplied with the AmpFlSTR® YfilerTM kit. The classification of Y-chromosome haplogroup for each male participant genotyped was done using haplogroup prediction program FTDNA 2.0 (http://www.hprg.com/hapest5/index.html).

#### Mitochondrial haplotype analysis

DNA samples were amplified by a single PCR to analyze the entire sub-regions HV1, HV2 and HV3 of mtDNA control region, using primers L15879 (5′-AATGGG CCTGTCCTTGTAGT-3 ′) and H727 (5′- AGGGTGAACTCACTGGAACG-3′). These primers were designed using the Primer3 program (http://www-genome.wi.mit.edu/cgi-bin/primer/primer3_www.cgi). PCR products were purified using EXO/SAP (Thermo Scientific Fermentas), and sequencing was done using the BigDye Terminator Cycle Sequencing Kit (Applied Biosystems) according to manufacturer's protocol. Capillary electrophoresis was performed using the ABI PRISM 3130 sequencer and resultant sequences analyzed using specific software BioEdit (http://www.mbio.ncsu.edu/BioEdit/BioEdit.html). The sequences obtained were compared with the Cambridge Reference Sequence (rCRS) (Andrews *et al.*, 1999) for final definition of haplotypes using the program Haplosite (http://www.haplosite.com/haplosearch/). Classification of mtDNA haplogroups was done using HaploGrep program (http://haplogrep.uibk.ac.at/).

### Statistical analysis

We evaluated the differences between the allele frequency of *STAT5B* c.424_427del mutation and the frequencies of each pathogenic allele identified in ExAC and ESP databases by using chi-square test. A p value less than 0.05 was considered statistically significant. All statistical calculations were carried out using SigmaStat version 3.5 (Systat Software Inc. Chicago, IL).

## Results

### *STAT5B* genotyping

Among the 1,205 evaluated individuals from the local population, seven were identified as heterozygous for *STAT5B* c.424_427del mutation. Two of them were later found to be 3^rd^ and 4^th^ degree relatives of the previously described homozygous patients (Figure 1). The other five were from four apparently unrelated families. Moreover, in the family who lost two children with a similar phenotype to the homozygous boys, the parents were first cousins and heterozygous for *STAT5B* c.424_427del mutation. Consequently, it is possible to infer that their deceased son and daughter were homozygous for this mutation and died of progressive pulmonary fibrosis.

In total, we identified seven apparently independent families with *STAT5B* c.424_427del mutation in Criciúma (Figure 1), with 33 heterozygous individuals. Therefore, the minor allelic frequency of this mutation at Criciúma was 0.29% (Confidence interval (CI) 95% of 0.08 to 0.5%). This allele frequency was significantly higher than the frequency of other pathogenic *STAT5B* allele variants observed in public databases (p < 0.001, Table 1 and Supplementary Table S1). Six of these families had ancestors born in Tubarão, a city located 60 km from Criciúma. In one family it was not possible to trace its geographic origin.

**Table 1:** Allele frequencies of pathogenic *STAT5B* variants in the present study and in public databases.

Database	Allele variant	Allele frequency (%)	Total alleles	p
Present study	17:40375522 TGGAG/T; p. Leu142Argfs*19	0.29	2,410	
ESP	17:40370236:C/T; p. Gln368*	0.008	13,006	< 0.0001
ExAC	17:40384025 G/A; p. Gln41*	0.001	121,406	< 0.0001
	17:40370235:T/TG; p. Gln368Profs*9	0.08	118,824	0.001
	17:40370235 TG/T; p. Gln368Argfs*2	0.03	118,824	< 0.0001
	17:40379567 G/GC; p. His89Alafs*11	0.001	121,240	< 0.0001

ESP: NHLBI-GO Exome Sequencing Project; ExAC: Exome Aggregation Consortium.

### Microsatellite analysis

Eleven different alleles were identified for D17S932 marker, ranging from 178 to 202 base pairs (bp), and nine alleles were identified for D17S1801 marker, ranging from 220 to 244 bp. The alleles 188 bp for D17S932 and 232 bp for D17S1801 were the most frequent alleles, with allele frequencies of 24% and 53%, respectively.

The analysis of these markers around *STAT5B* showed that the two homozygous patients had the haplotype D17S932 196, D17S1801 242. Both markers were identified in all heterozygous individuals (100%) and their phase has been inferred from families with unambiguous haplotypes. However, this combination of markers was found in only 5 of 53 evaluated non-carriers (9.4%), which was significantly different from heterozygous carriers (considering one individual per family, p < 0.001). Based on this result, it is unlikely that *STAT5B* c.424_427del mutation associated with 196-242 haplotype was derived from independent origins. The alternative hypothesis is that individuals harboring the mutation share a common ancestor, from whom they inherited this haplotype.

### Ancestry analysis

The ancestry in all heterozygous subjects evaluated (one from each affected family) was predominately assigned to a cluster defined by its maximum frequency in European populations (median 85%, p25-p75 80.9-89.3%). African and Amerindian ancestries represented 7.4% (p25-075 3.1-9.1%) and 7.2% (p25-p75 6.5-14%) of the genomes analyzed (Table 2).

**Table 2:** Summary of genomic ancestry analysis and Y-chromosome and mitochondrial DNA haplogroups.

Family	Genomic ancestry			Y-chromosome haplotype	Geographic origin	Mitochondrial haplotype	Geographic origin
	European	Amerindian	African				
1	0.850444	0.149546	0.00001	J2a1	Middle East	H	Europe
2	0.866579	0.059787	0.073634	R1b	Western Europe	C1	Amerindian
3	0.932712	0.013584	0.053705	R1b	Western Europe	H	Europe
4	0.843647	0.06985	0.086503	female		B4	Amerindian
5	0.918863	0.07191	0.009226	I2a1	Middle East	B4	Amerindian
6	0.774306	0.130023	0.095671	J1	Middle East	HV	Europe
7	0.650728	0.164811	0.184461	R1b	Western Europe	K1a	Northwest Europe

Regarding the paternal lineage origin, the haplogroup R1b, which is predominantly found in Western Europe, was present in three of them. Two of them had the haplogroup J (J1 e J2a1), found mainly in the Middle East, and one individual had the rare haplogroup I2a1 (Table 2). About the maternal lineage origin, the haplotypes B4 and C1, found in indigenous peoples of the Americas, were present in three subjects. The haplotype H, the most common maternal lineage in Europe, was found in two other individuals. One subject had the haplotype K1a, present in all Europe but more prevalent in Northwest Europe, and the last one had the haplotype HV, present in the Middle East, Italy and Eastern Europe.

## Discussion

Growth hormone insensitivity with immune dysfunction (OMIM 245590) caused by *STAT5B* mutations is a rare but life threatening disease in which severe short stature is accompanied by the burden of recurrent infections, autoimmune diseases and progressive pulmonary fibrosis. Four out of 10 described patients died of respiratory failure, including one of them who was submitted to lung transplantation when he was 17.5 years old. There is no treatment available at present, but some researchers consider that early bone marrow transplantation could prevent the pulmonary disease. Inheritance is autosomal recessive and none of the described patients had compound heterozygous mutations. Family history was available in eight cases and consanguinity was not identified in half of them (Bernasconi *et al.*, 2006; Vidarsdottir *et al.*, 2006; Pugliese-Pires *et al.*, 2010).

Regarding the Brazilian family, the absence of consanguinity and the finding of another affected family in the same city motivated us to investigate the frequency of *STAT5B* c.424_427del mutation in the region of Criciúma. We found that *STAT5B* c.424_427del allele frequency in this region is 0.29%, more than three times higher than the frequency of the most frequent pathogenic allele variant identified in public population databases (Table 1). Considering this mutation frequency and the birth rate at Criciúma (according to 2013 IBGE census), it is possible to estimate the incidence of new homozygous cases as 1 every 40 years. However, if we consider the highest possible incidence according to the confidence interval, this incidence may reach 1 every 13 years.

The presence of the same haplotype in all evaluated *STAT5B* c.424_427del mutation carriers and the relative rarity of the same haplotype in non-carrier individuals from the same region indicate a common origin of this mutation and the existence of a founder effect justifying its relatively high prevalence in Criciúma. Studies analyzing other defects in GH/IGF1 axis presented similar findings, in which a founder effect explained the presence of the same mutation in families apparently not related (Kamijo *et al.*, 2004).

Criciúma was founded in 1880 with the arrival of 22 families of Italian immigrants from the Veneto region, which was followed by the arrival of Portuguese, German and Polish immigrants. It was originally a part of Tubarão, the city where most families with *STAT5B* c.424_427del mutation had their origin. Tubarão was first occupied in 1774 by Portuguese and Azoreans immigrants and Brazilians of mixed ancestry; it then received Italian, German and Polonese immigrants at the end of the 19^th^ century.

The genomic ancestry analysis indicated a European origin of this mutation. The Y-chromosome haplotype analysis showed Western European and/or Middle East origin of *STAT5B* c.424_427del mutation. This finding suggests a probable Portuguese/Spanish origin, although it is not possible to rule out completely an Italian origin of this mutation. The maternal lineage origin analysis by mtDNA presented both European and Amerindian haplogroups, showing the existence of marriages among European descendants, but also between them and Native Americans, as observed in other Latin American populations (Alves-Silva *et al.*, 2000).

In conclusion, our findings provide evidence to the existence of a founder effect explaining a relatively elevated prevalence of *STAT5B* c.424_427del mutation in the south of Brazil. According to Y-chromosome haplotype analysis, we hypothesize that Portuguese immigrants or descendants carried this mutation to Tubarão and from there to Criciúma. Health professionals from Criciúma and its neighboring cities, including Tubarão, should be aware of the increased risk of homozygosity for this mutation in the region so that new cases are early diagnosed.

## Supplementary material

The following online material is available for this article:

Table S1 - Allele frequency comparison of the present study with ExAC data.
