# Supplementary material for: Growth hormone insensitivity with immune dysfunction caused by a STAT5B mutation in the south of Brazil: evidence for a founder effect
Source: Genet Mol Biol. 2017 Jun 5;40(2):436–41. doi: 10.1590/1678-4685-GMB-2016-0231 (PMC5488464; doi:10.1590/1678-4685-GMB-2016-0231)
Supplement: Supplementary file 1 [file 1415-4757-gmb-1678-4685-GMB-2016-0231-Suppl01.pdf]

Table S1 - Comparison of the allele frequency found in the present study (0.29%) with allele frequencies of pathogenic *STAT5B* variants found in Exome Aggregation Consortium (ExAC) database according to population.

	European		African		Latino	
Allele variant	Allele frequency (%)	Total alleles	Allele frequency (%)	Total alleles	Allele frequency (%)	Total alleles
17:40384025 G/A	0	66734	0	10406	0.009	11578
17:40370235:T/TG	0.109	65222	0.158	10132	0.009	11350
17:40370235 TG/T	0.035	65222	0.049	10132	0.053	11350
17:40379567 G/GC	0.002	66654	0	10354	0	11562
